# Variables associated with pacemaker implantation in postoperative patients of cardiac surgery in a university hospital

**DOI:** 10.1186/2197-425X-3-S1-A958

**Published:** 2015-10-01

**Authors:** J Escobar, A Archila, L Delgado, A Leon, H Atehortua, F Jaimes

**Affiliations:** Universidad de Antioquia, Medellin, Colombia; Universidad de Antioquia, Internal Medicine, Medellin, Colombia

## Introduction

In postoperative cardiac patients, the incidence of conduction disturbances is between 1 to 4% depending on the complexity of the surgical procedure, the intraoperatory complications and the need for reintervention. These disorders lead to the need for electrical support, transient or permanent by a pacemaker. So far, there are few data in the literature regarding the potential factors related to the need for a pacemaker in postoperative cardiac surgery.

## Objectives

To determine which clinical or surgical variables are associated with the need for a pacemaker in postoperative cardiac surgery.

## Methods

Retrospective cohort with consecutive clinical records of patients in postoperative cardiac surgery from January 2006 through June 2011 at the University Hospital, San Vicente Foundation (Medellín, Colombia). The following variables were evaluated regarding their relationship with the requirement for a pacemaker using a multiple logistic regression model: ejection fraction, preoperative functional class, arrhythmia at hospitalization, time of perfusion, and the value of the EUROSCORE.

## Results

From 760 clinical records evaluated, 73 required pacemaker during or after surgery. The mean age was 54 years (SD = 17); 58% (443) were men; the mean ejection fraction was 54% (SD = 15); 40% (n = 309) had functional class III or IV, and half (n = 378) had previous valvular disease. The mean pump time was 104 minutes (SD = 38) and the EUROSCORE was 6 (SD = 3). In the multivariate model, none of the evaluated variables were associated with the need for a pacemaker: ejection fraction (OR = 0.98; CI 95% = 0.96 - 1.01); arrhythmia (OR = 1.45; CI 95% = 0.56 - 3.75); perfusion time (OR = 1.00; IC 95% = 0.99 - 1.01); EUROSCORE (OR = 0.99; CI 95 = 0.92 - 1.06) and functional class (all OR with confidence intervals through the unit).

## Conclusions

In our study population, none of the variables, previously reported in the literature, showed association with the pacemaker requirement. Although in the univariate analysis previous arrhythmias and functional class show a tendency to be associated, it is not possible to determine if the inconclusive results are due to sample size limitations or to undefined particularities of our population.

